# Supplementary material for: Redox Metabolism Measurement in Mammalian Cells and Tissues by LC-MS
Source: Metabolites. 2021 May 13;11(5):313. doi: 10.3390/metabo11050313 (PMC8153172; doi:10.3390/metabo11050313)
Supplement: Supplementary file 1 [file metabolites-11-00313-s001.zip › Figure S4. Application of the redox metabolite detection method for profiling redox state following pharmacologic perturbations of redox balanc.pdf]

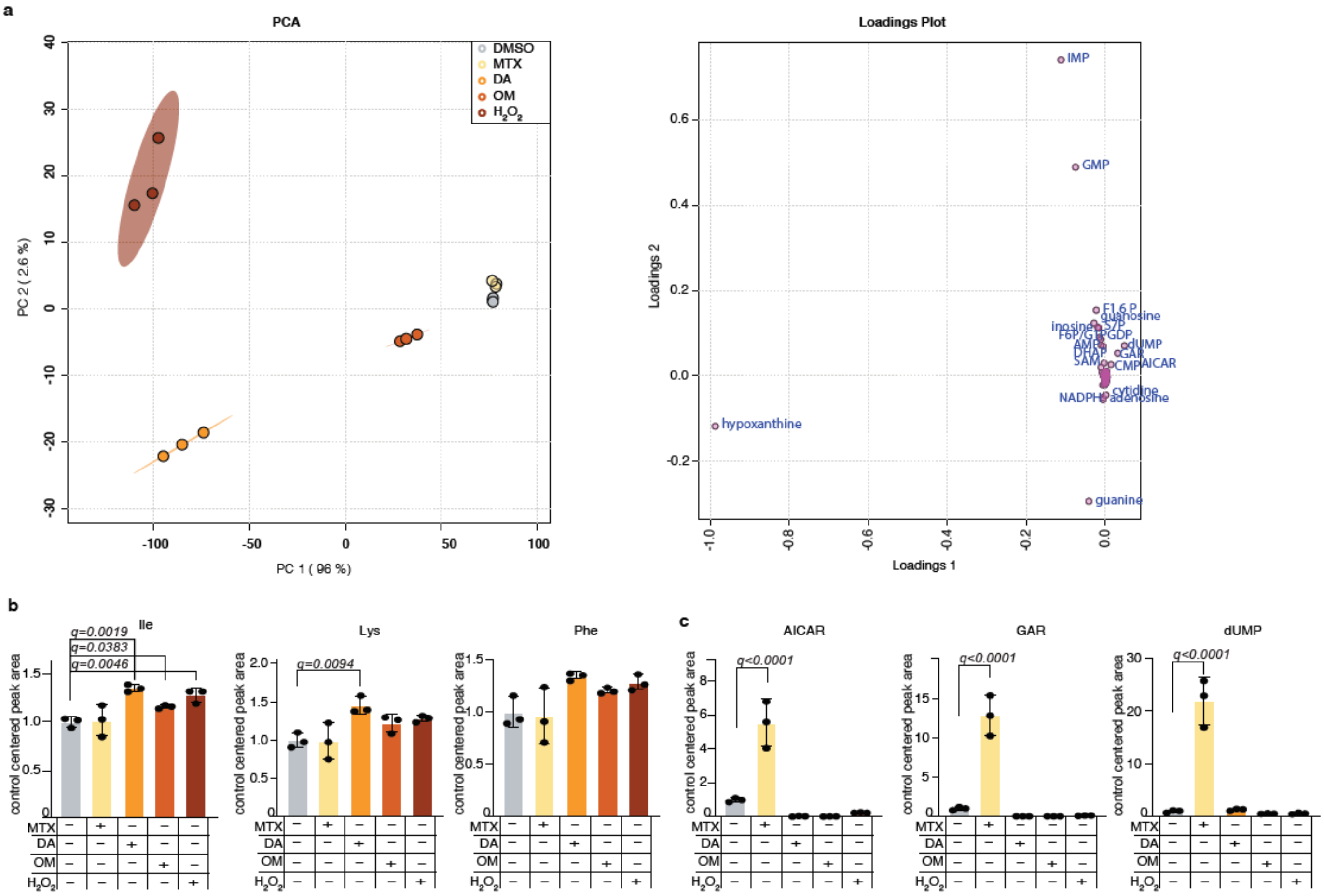

Figure 4. Application of the redox metabolite detection method for profiling redox state following pharmacologic perturbations of redox balance in cells (**a**) K562 cells were treated with drugs that perturb the cellular redox balance, using the indicated doses for 4h. PLSDA and corresponding loading plot are presented. Important metabolites contributing to groups separation are indicated; (**b**) Amino acid levels upon treatment with redox-perturbing drugs are indicated; statistical significance was determined using Anova with correction for multiple comparisons and false discovery rate. Only significant q-values are indicated; (**c**) Methotrexate triggers expected changes in nucleotides and nucleotide synthesis intermediates. Presented are the purine synthesis pathway intermediates AICAR and GAR, as well as the nucleotide dUMP. Mean and standard deviation of one experiment performed in triplicate; statistical significance was determined using Anova with correction for multiple comparisons and false discovery rate. Only significant q-values are indicated.
