# Supplementary material for: Redox Metabolism Measurement in Mammalian Cells and Tissues by LC-MS
Source: Metabolites. 2021 May 13;11(5):313. doi: 10.3390/metabo11050313 (PMC8153172; doi:10.3390/metabo11050313)
Supplement: Supplementary file 1 [file metabolites-11-00313-s001.zip › Figure S2. Optimization of redox metabolite detection in mammalian cells.pdf]

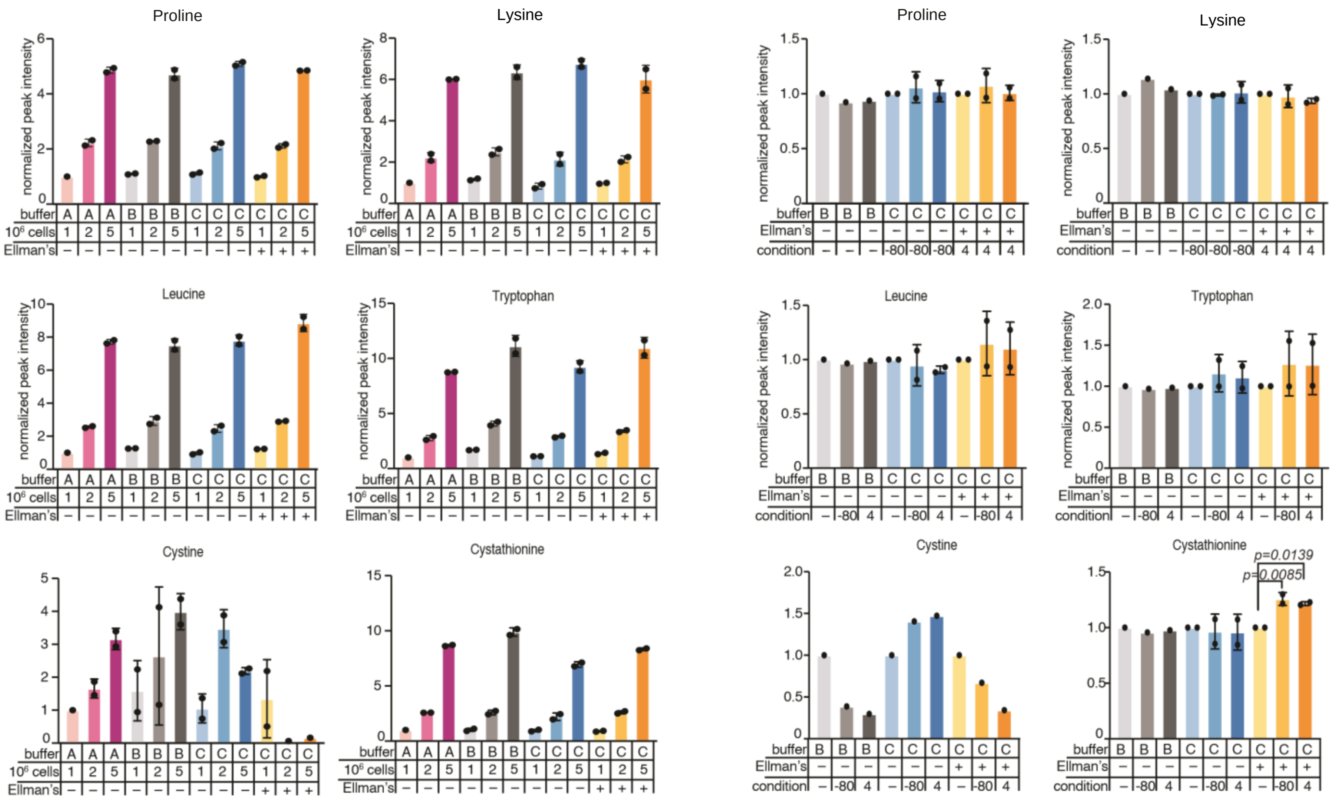

Figure S2. Optimization of redox metabolite detection in mammalian cells (**a**) Comparison of metabolite detection in samples extracted from the mammalian cell line K562 using three extraction buffers (A, B, and C) with increasing number of cells (1, 2, or 5 million cells). Values were normalized to Buffer B/1Million cells condition and present the average values and standard deviation of at least two independent experiments each with technical triplicates (except for buffer A, 1M condition, which was measured once with a technical duplicate; (**b**) Comparison of metabolite detection in samples extracted Figure 562. and detected following the three storage conditions: "-": extraction followed by LC-MS analysis immediately upon harvest, "-80": analysis following storage of the sample in -80C° for 24 hours, and condition "4" that included a second incubation of the sample at 4C° for 24 hours before analysis by LC-MS. Values were normalized for each buffer to the corresponding "-" condition that involved no long storage. Presented are the average values and standard deviation of two independent experiments each with technical triplicates.
