# Supplementary material for: Redox Metabolism Measurement in Mammalian Cells and Tissues by LC-MS
Source: Metabolites. 2021 May 13;11(5):313. doi: 10.3390/metabo11050313 (PMC8153172; doi:10.3390/metabo11050313)
Supplement: Supplementary file 1 [file metabolites-11-00313-s001.zip › Figure S3. Application of the redox metabolite detection method for mammalian tissues.pdf]

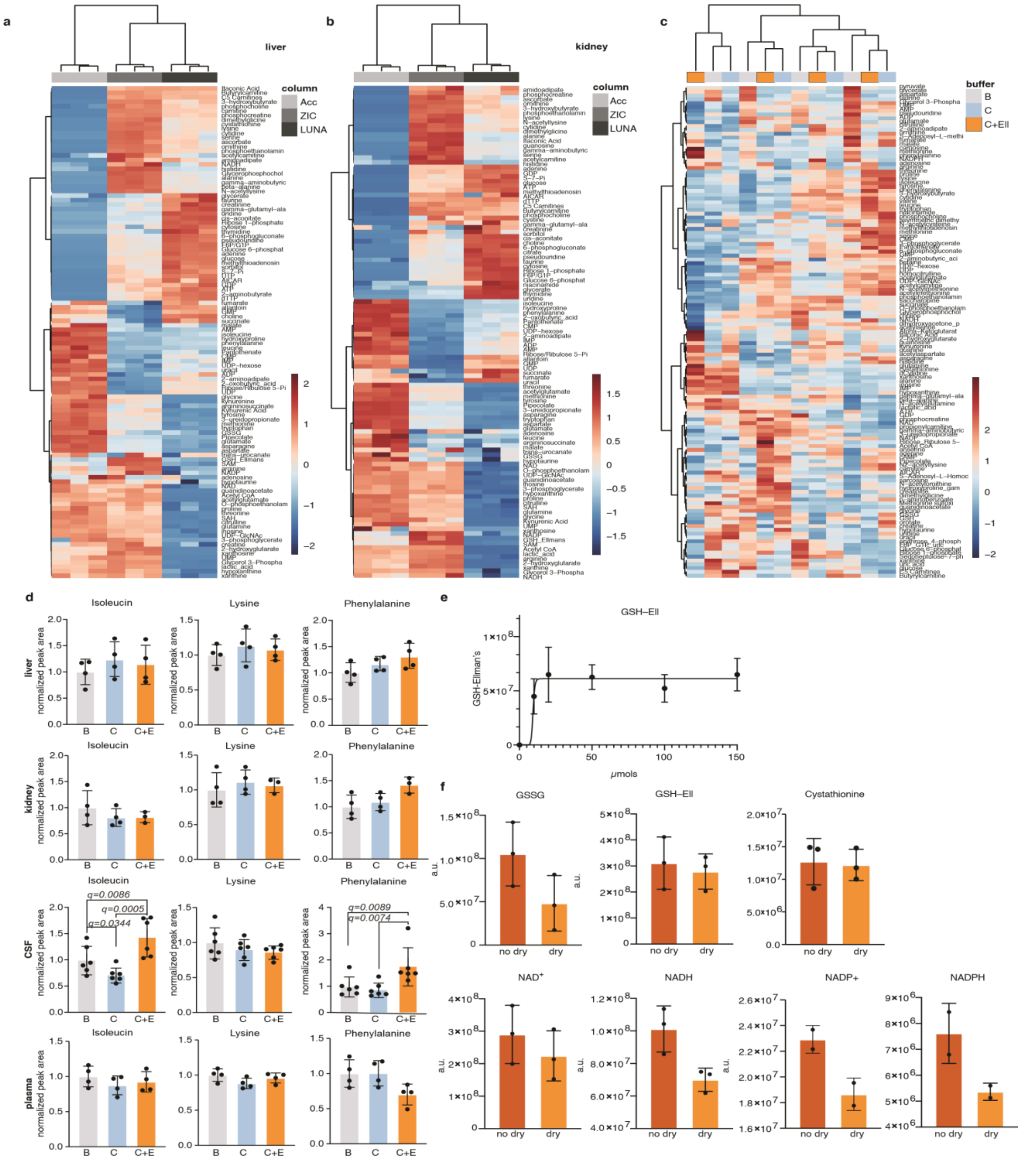

Figure S3. Application of the redox metabolite detection method for mammalian tissues; (**a**,**b**) LC-MS analysis of mouse liver (**a**) and kidney (**b**) using three chromatographic methods coupled to MS. Polar metabolites are presented in a heatmap for each organ; (**c**) Heatmap cluster analysis of polar metabolites detection in mouse liver samples extracted with three different buffers (B, C, C plus Ellman's); (**d**) Amino acid levels in the mouse liver, kidney, CSF, and plasma as detected by LC-MS following extraction in three buffers: B, C, C plus Ellman's; statistical significance was determined using Anova with correction for multiple comparisons and false discovery rate. Only significant q-values are indicated (except for GSH and GSH-Ell levels, which were excluded); (**e**) A big chunk of mouse liver was extracted in buffer C, split among indicated conditions and different concentrations of Ellman's reagent were added immediately. Raw peak areas for GSH-Ell were integrated and a non-linear model was used to fit the data. Presented are the average values and standard deviation of at least two independent experiments; (**f**) Mouse liver portions were extracted in buffer containing Ellman's reagent and split in half. One set of samples was dried before running for detection by LC-MS, while the other set was immediately analyzed by LC-MS, without prior drying and resuspension of the sample in water. Presented are the average values and standard deviation of three biological repeats.
