# Supplementary material for: Redox Metabolism Measurement in Mammalian Cells and Tissues by LC-MS
Source: Metabolites. 2021 May 13;11(5):313. doi: 10.3390/metabo11050313 (PMC8153172; doi:10.3390/metabo11050313)
Supplement: Supplementary file 1 [file metabolites-11-00313-s001.zip › FIgure S1. Optimization of redox metabolites detection by LC-MS.pdf]

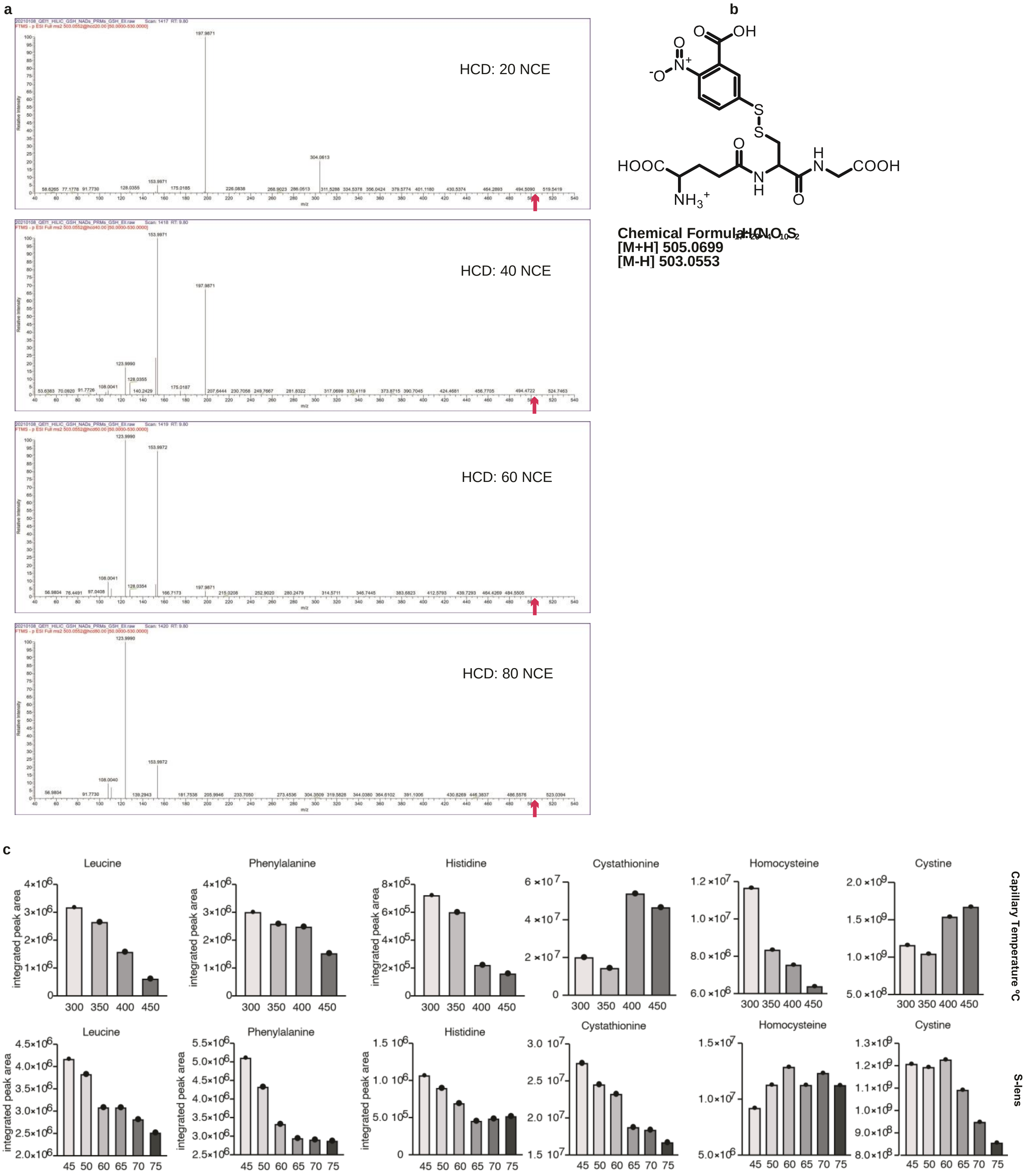

Figure S1. Optimization of redox metabolites detection by LC-MS (**a**) MS2 spectra for the indicated normalized collision energies (NCE) of the derivatization product of Ellman's with glutathione (GSH-Ell). Red arrow indicates mass of precursor; (**b**) Schematic of the chemical structure and theoretical exact mass of negative of positive ion of GSH-Ell; (**c**) Optimization of HESI parameters on orbitrap mass spectrometer for the indicated amino acids using ZIC-pHILIC chromatography and 13C15N isotopically labeled standards. Graphs represent integrated areas of chromatographic peaks under changing parameters for capillary temperature or S-lens.
